# A transgenic inducible GFP extracellular-vesicle reporter (TIGER) mouse illuminates neonatal cortical astrocytes as a source of immunomodulatory extracellular vesicles

**DOI:** 10.1038/s41598-019-39679-0

**Published:** 2019-02-28

**Authors:** Victoria N. Neckles, Mary C. Morton, Jennie C. Holmberg, Aidan M. Sokolov, Timothy Nottoli, Don Liu, David M. Feliciano

**Affiliations:** 1 Department of Biological Sciences, Clemson University, Clemson, SC 29634-0314 USA; 2 Yale Genome Editing Center, Department of Comparative Medicine, Yale School of Medicine, PO Box 208016, New Haven, CT 06520-8016 USA; 3 Charles River Laboratories, 261 Ballardvale Street, Wilmington, MA 01887 USA

## Abstract

Extracellular vesicles (EVs) are cellular derived particles found throughout the body in nearly all tissues and bodily fluids. EVs contain biological molecules including small RNAs and protein. EVs are proposed to be transferred between cells, notably, cells of the immune system. Tools that allow for *in vivo* EV labeling while retaining the ability to resolve cellular sources and timing of release are required for a full understanding of EV functions. Fluorescent EV fusion proteins are useful for the study of EV biogenesis, release, and identification of EV cellular recipients. Among the most plentiful and frequently identified EV proteins is CD9, a tetraspanin protein. A transgenic mouse containing a CRE-recombinase inducible CAG promoter driven CD9 protein fused to Turbo-GFP derived from the copepod *Pontellina plumata* was generated as an EV reporter. The transgenic inducible GFP EV reporter (TIGER) mouse was electroporated with CAG-CRE plasmids or crossed with tamoxifen inducible CAG-CRE-ER^T2^ or nestin-CRE-ER^T2^ mice. CD9-GFP labeled cells included glutamine synthetase and glial fibrillary acidic protein positive astrocytes. Cortical astrocytes released ~136 nm EVs that contained CD9. Intraventricular injected EVs were taken up by CD11b/IBA1 positive microglia surrounding the lateral ventricles. Neonatal electroporation and shRNA mediated knockdown of Rab27a in dorsal subventricular zone NSCs and astrocytes increased the number of CD11b/IBA1 positive rounded microglia. Neonatal astrocyte EVs had a unique small RNA signature comprised of morphogenic miRNAs that induce microglia cytokine release. The results from this study demonstrate that inducible CD9-GFP mice will provide the EV community with a tool that allows for EV labeling in a cell-type specific manner while simultaneously allowing *in vivo* experimentation and provides evidence that EVs are required immunomodulators of the developing nervous system.

## Introduction

Extracellular vesicles (EVs) are nanometer sized particles that are released from numerous central nervous system cell types and are implicated in a wide range of neurological diseases^1^. Work focused on brain development has identified EVs within fetal, perinatal, and adult cerebrospinal fluid (CSF)^2,3^. One source of EVs are neonatal subventricular zone (SVZ) neural stem cells (NSCs)^4^. NSC EVs act as an immunomodulator that are taken up by CD11b/IBA1 positive immune cells in the SVZ during perinatal development^4,5^. NSC EVs facilitate transcriptional network re-wiring of microglia and subsequent release of cytokines.

SVZ NSCs begin to diminish and recede in number as rodents age^6^. In humans, SVZ NSCs exhaust by 18 months of age^7^. Although the embryonic brain is colonized by microglia early in embryonic development, a band of microglia appears within the ventricular zone near the end of neurogenesis, and a population of CD11b positive microglia localizes to the postnatal rodent SVZ^8–11^. As neurogenesis slows and dorsal SVZ NSCs generate astrocytes, many SVZ microglia disperse^8,12^. This coincides with the dispersion, activation state conversion, and morphological maturation of microglia. The mechanisms responsible for microglia dispersion and maturation are unclear.

A mechanism that could partially account for changes in microglia is that sources of EVs also change. As the number of SVZ NSCs decreases, fewer NSC EVs are produced. Around the same time, lower cortical layer astrocytes are produced from dorsal SVZ progenitors and upper cortical layer astrocytes are produced by additional progenitors^12^. Perinatal astrocytes could in theory be an additional EV source. In fact, SVZ NSCs are a special type of astrocyte, they are SVZ astrocytes^13^. SVZ NSCs and cortical astrocytes also share cellular ontogeny and overlap in biochemical markers^13^. Moreover, astrocytes can generate neurons upon transplantation to a permissive environment, for example, the SVZ^14^. Upon introduction of specific transcription factors, for example SOX2, astrocytes become neurogenic^15^. Predictably, during the neonatal period, astrocytes, particularly reactive astrocytes, are released from their gliogenic fate potential^14^.

EV studies are commonly performed *in vitro* because there are few tools to study EVs *in vivo* and therefore evidence of elaborate hypotheses requires further substantiation. *In vivo* electroporation of DNA plasmids that encode for fluorescent EV labels has at least in part addressed this issue^4,16^. However, there remains considerable limitation regarding the cell types and timing of EVs labeled which prevents our understanding of the morphogenic nature of EV signals. Here, a solution to this problem is presented in the form of a transgenic inducible GFP extracellular-vesicle reporter (TIGER) mouse. The use of this mouse provides evidence that astrocytes produce CD9 positive EVs and that these EVs have immunomodulatory and morphogenic properties.

## Results

CD9 is enriched in EVs and therefore is frequently described as an EV marker protein. The generation of a transgenic inducible and fluorescently tagged CD9 (TIGER) mouse would facilitate *in vivo* identification of EV sources and target cells. A CRE inducible carboxy terminal His-tagged CD9-GFP targeting plasmid was generated to control the cell types and timing of CD9-GFP expression, (Supplemental Fig. 1A). The plasmid contains a lox-STOP-lox sequence upstream of CD9-GFP. When CRE recombinase is co-expressed, the stop sequence will be deleted and CD9-GFP expression will then be controlled by the CAG promoter. This plasmid contains flanking sequences for targeting to the ROSA locus and a neomycin resistance (NeoR) cassette for G418 selection in ES cells. To confirm system functionality, Neuro2a cells co-transfected with CD9 and CAG-CRE plasmids expressed CD9-GFP (Supplemental Fig. 1B). Targeting in mouse embryonic stem (ES) cells was performed essentially as described^17^. The targeting plasmid was electroporated into S1B6A ES cells, derived from F1 hybrid B6;129S1 embryos. 24 hr post-electroporation, cells were cultured in the presence of G148. 120 surviving clones were screened for CD9-GFP and the NeoR cassette (Supplemental Fig. 1C and data not shown). As would be predicted, all cells contained the NeoR cassette based on their ability to survive G148 (data not shown). 24 ES clones were screened using a long arm assay first by long range PCR and four positive clones were identified (Supplemental Fig. 1C). Then the remaining two plates were screened, including the four positive clones as controls. Additional positive clones were identified. Since the long-range PCR assay is not robust, and may give false negative results, we tested eight putative positive clones using a 3′ end assay (Supplemental Fig. 1D). Three ES clones were identified as properly targeted. Positive ES clones were microinjected into C57Bl/6 J blastocysts and injected embryos transferred into pseudopregnant CD1 mice. High percentage chimeras were mated with C57Bl/6 J mice and germline transmission of the targeted allele (TIGER) was confirmed in the G1 pup (Supplemental Fig. 1E,F,G).

CD1 mice were subjected to neonatal subventricular zone electroporation with CD9-GFP (green) and fluorescent tdTomato (red, not shown) to examine CD9 subcellular distribution (Supplemental Fig. 2A–C)^16^. Next, TIGER mice were crossed with inducible Tomato^+/+^ mice and P0 pups were subjected to neonatal electroporation with CAG-CRE plasmid (Supplemental Fig. 2D). Brains were harvested two days post-electroporation, sectioned, and counterstained with the DNA dye TO-PRO-3. Confocal microscopy revealed CD9-GFP fluorescence indicative of *in vivo* recombination in a fraction of SVZ cells (Supplemental Fig. 2E–G). This is consistent with previous quantifications of CD9-GFP and tdTomato electroporated mice that demonstrated CD9-GFP was expressed in ~27% of all electroporated cells^4^. Taken together CRE induces CD9-GFP in TIGER mice.

**Figure 1:**
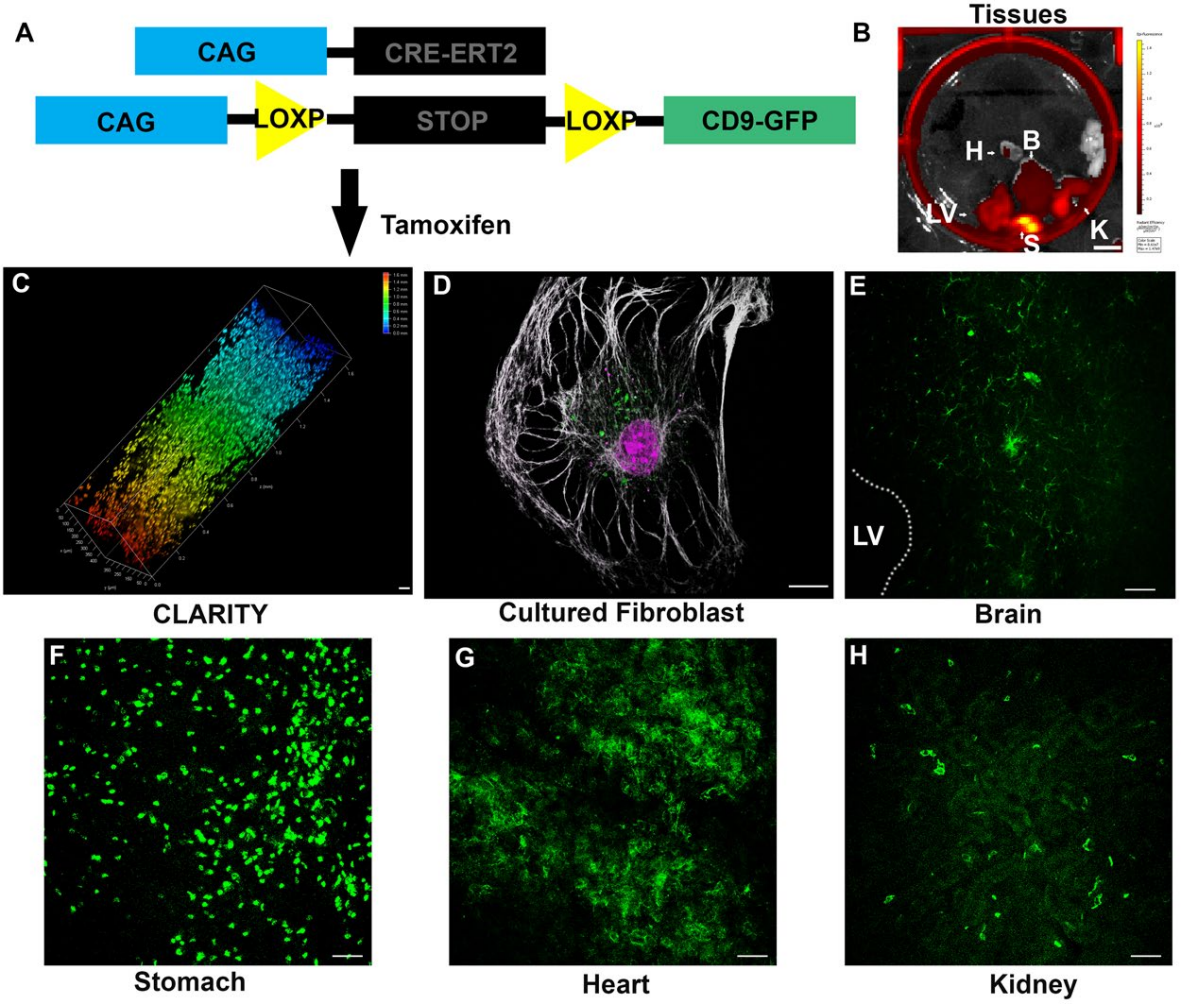
(**A**) Schematic of mouse genotype. (**B**) Image of dissected tissue from CAG-CRE-ER^T2^ × TIGER mouse cross following 100 μg/g tamoxifen injection at P2 and sacrificed at P4. H, heart, B, brain, LV, liver, S, stomach, K, Kidney. Scale bar, 1 cm. (**C**) Depth coded volumetric 2 photon microscopy image of CD9-GFP following 100 μg/g tamoxifen injection starting at P2 until sacrifice at P8 in a spleen following CLARITY. Color indicates depth of imaging. Scale bar, 50 μm. (**D**) Cultured fibroblast treated with tamoxifen from TIGER × CAG-CRE-ER^T2^ mice. Scale bar, 12.5 μm (**E**–**H**) Sections of TIGER × CAG-CRE-ER^T2^ mouse tissue following 100 μg/g tamoxifen at P2 and sacrificed at P4. (**E**) Coronal section of TIGER × CAG-CRE-ER^T2^ mouse near ventricle showing CD9-GFP positive cells. Scale bar, 25 μm. (**F**) Section of stomach of a TIGER × CAG-CRE-ER^T2^ mouse. Scale bar, 25 μm. (**G**) Section of a heart of a CD9-GFP × CAG-CRE-ER^T2^ mouse. (**H**) Section of kidney of a TIGER × CAG-CRE-ER^T2^ mouse. Scale bar, 25 μm.

TIGER mice were next crossed to CAG-Cre-ER^T2^ mice and injected with 100 μg/g tamoxifen from P2 until they were sacrificed at P8 (Fig. 1A)^18^. CAG-CRE-ER^T2^ activation is limited in the brain after birth, is dose dependent, and time dependent^18^. Fluorescence was detected by IVIS imaging within a wide range of tissues including the stomach, kidney, heart, brain, liver, and lung (Fig. 1B)^18^. CD9-GFP fluorescence was confirmed by CLARITY and whole tissue imaging with two-photon microscopy (Fig. 1C). The expression and distribution of CD9-GFP within cells was examined in cultured fibroblasts. CD9-GFP expression was induced only when cells were cultured with tamoxifen (Fig. 1D). Tissues were sectioned, subjected to immunohistochemistry, and imaged by confocal microscopy and CD9-GFP expression was confirmed within each tissue (Fig. 1E–H). The results from these experiments demonstrate tissue and cell-type dependent recombination and CD9-GFP expression.

TIGER mice were crossed with Nestin-Cre-ER^T2^ and Tomato^+/+^ mice and tamoxifen given three times at P8 and sacrificed at P11 which corresponds to periods of peak astrogliogenesis (Supplemental Fig. 3A–G)^19^. CD9-GFP was detected in cells with an astrocyte-like morphology in the cerebral cortex following tamoxifen injection (Supplemental Fig. 3C–G). The results from these experiments demonstrate that CD9-GFP expression is specific when using a highly selective promoter driven CRE system and confirm that recombination occurs in cells co-expressing tomato.

**Figure 2:**
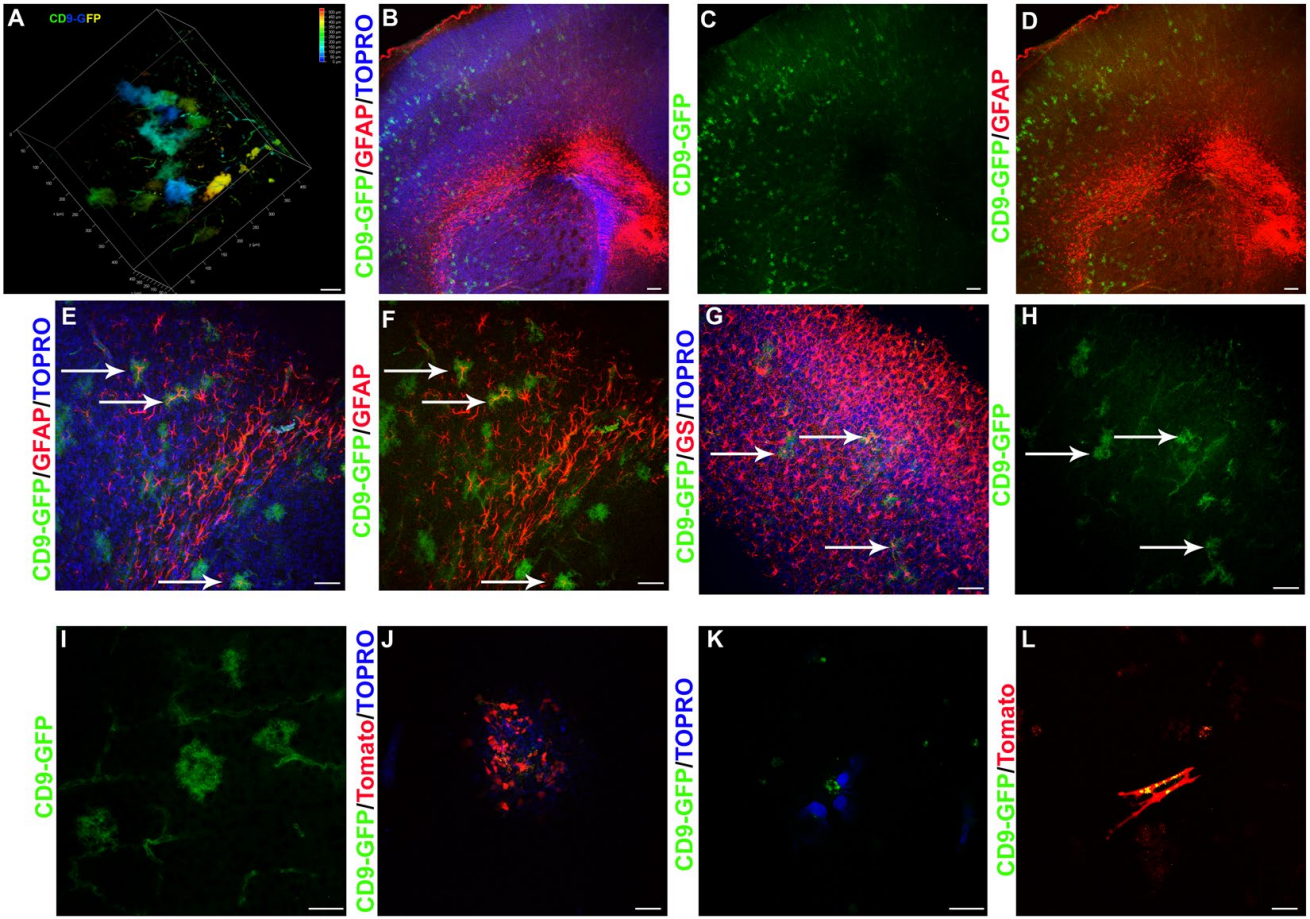
(**A**) Depth coded volumetric rendering of cerebral cortex of a TIGER × Nestin-CRE-ER^T2^ mouse. Scale bar, 50 μm. (**B**–**D**) CD9-GFP (green) × CAG-CRE-ER^T2^ brain sectioned and stained for GFAP (red) and counter-stained with TOPRO (blue) to label nuclei merged (**B**), CD9-GFP alone (**C**), and CD9-GFP/GFAP (**D**). Arrows point to cells with co-localization. Scale bar, 100 μm. (**E**,**F**) 20X image of CD9-GFP (green) CAG-CRE-ER^T2^ brain sections stained for GFAP (red) and counter-stained with (**E**) or without (**F**) TOPRO (blue). Arrows point to colocalization. Scale bar, 50 μm. (**G**,**H**) Image showing CD9-GFP (green) from TIGER × CAG CRE ER^T2^ brain sections stained for GS (red) and TOPRO (blue) (**G**) or CD9-GFP only (**H**). Arrows points to colocalization. Scale bar, 50 μm. (**I**) 63X image of cortex demonstrating CD9-GFP in astrocytes. Scale bar, 25 μm. (**J**) Astrocyte culture from TIGER × Tomato mice electroporated with CRE and counter-stained with TOPRO (blue). Scale bar, 50 μm. (**K**) Cultured astrocytes from TIGER mice and counter-stained with TOPRO (blue). Scale bar, 12.5 μm. (**L**) CD9-GFP (green) and tomato (red) electroporated astrocyte cultures to demonstrate morphology. Scale bar, 25 μm.

Brains from tamoxifen injected CAG-CRE-ER^T2^ × TIGER mice were subjected to CLARITY and two-photon microscopy. CD9-GFP labeled cells in the cortex were reminiscent of the cortical astrocyte-like cells seen in TIGER × Nestin-CRE-ER^T2^ × Tomato^+/+^ mice (Fig. 2A). To determine the identity of these cells, brains were subjected to immunohistochemistry for the astrocyte marker protein GFAP which is expressed in a small population of astrocytes around the corpus callosum and SVZ under resting conditions. CD9-GFP and GFAP co-localization was found in lower cortical layers near the corpus callosum (Fig. 2B–F). All CD9-GFP positive cortical cells were also positive for the astrocyte marker, Glutamine Synthetase (GS) (Fig. 2G,H). High magnification highlighted the astrocyte-like morphology of these cells (Fig. 2I). The ability of astrocytes to express CD9 was confirmed by culturing TIGER × Tomato^+/+^ mice astrocytes electroporated with CRE, by culturing astrocytes from TIGER × Nestin-CRE-ER^T2^ mice injected with tamoxifen, and by CD9-GFP and tomato electroporation of astrocytes (Fig. 2J–L). These results demonstrate that astrocytes can express CD9.

**Figure 3:**
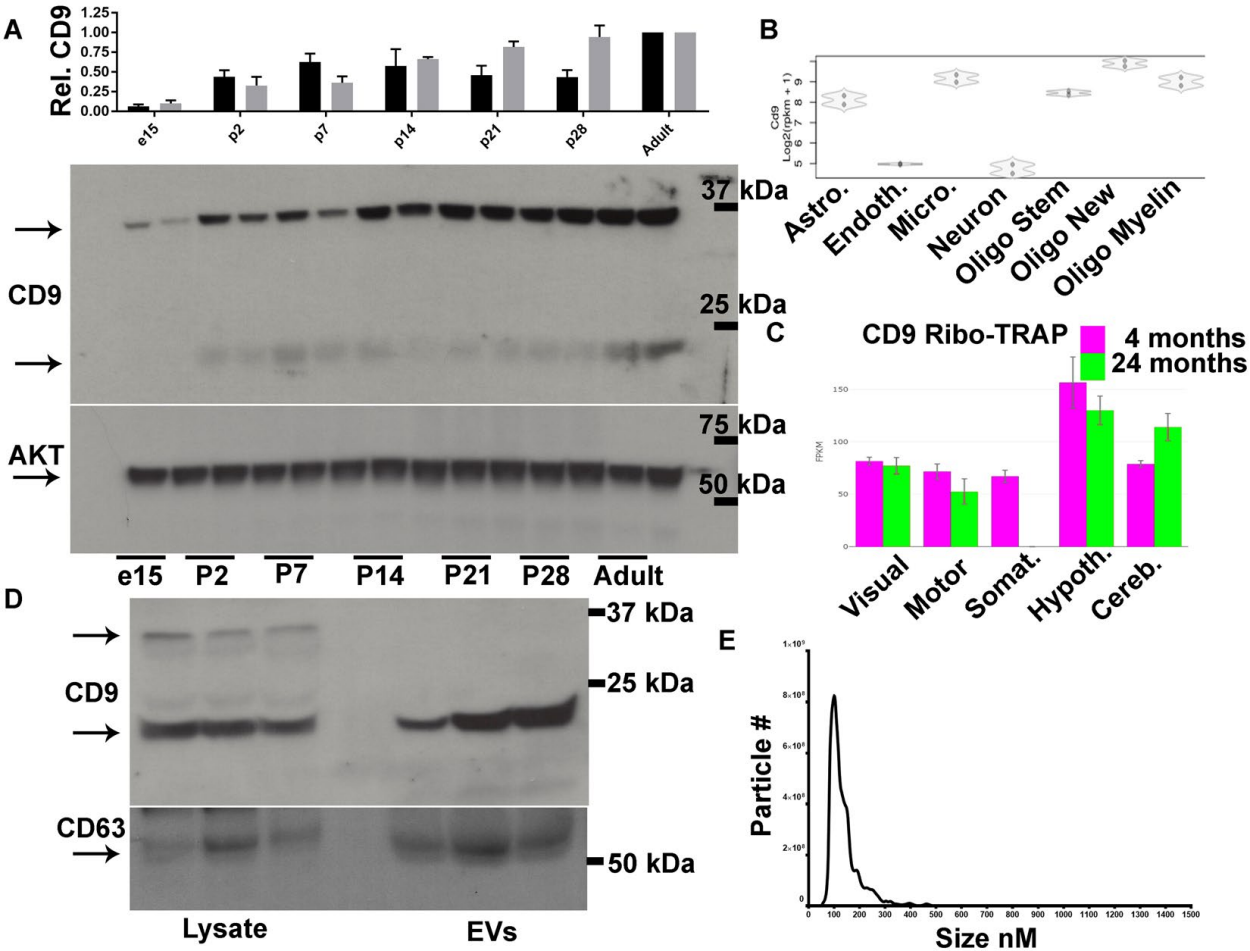
(**A**) Quantification of CD9 protein relative to AKT in cortical lysates at the indicated ages according to western blots of dorsal cortical lysates for CD9 and AKT as a loading control. The ~37 kDa (black bar) and 20–25 kDa (grey bar) CD9 bands were quantified. N = 3 independent biological replicates. (**B**) RNA sequencing reads for CD9 based on cell type listed. Astro. = Astrocyte, Endoth. = Endothelial cell, Micro = microglia, Oligo Stem = Oligodendrocyte stem cell, Oligo New = immature oligodendrocyte, Oligo Myelin = Mature myelinating oligodendrocyte. (**C**) Quantification of astrocyte CD9 mRNA associated with active ribosomes from different regions and ages. Visual = visual cortex, Motor = Motor cortex, Somat. = Somatosensory cortex, Hypoth. = Hypothalamus, Cereb. = Cerebellum. (**D**) Western blots of astrocyte lysate and EVs for CD9 and CD63. (**E**) Nanosight particle tracking analysis frequency histogram of astrocyte conditioned media. Data are represented as mean ± SEM.

It was unclear whether CD9-GFP expression in astrocytes was indicative of their potential to release EVs or was based on the absorption, distribution, metabolism, and excretion of tamoxifen as well as cell type specific expression of CAG-CRE-ER^T2^. Cortical CD9 expression was determined using two antibodies to CD9 and reliably detected bands between 20–25 kDa and ~37 kDa that increased in prevalence from e15 to adulthood (Fig. 3A, data not shown). To confirm that astrocytes express CD9 we performed bioinformatic analysis using two databases and validated CD9 mRNA expression in astrocytes by single cell and by ribotag RNA-sequencing (Fig. 3B,C)^20,21^. EVs from neonatal cortical astrocytes were collected from cultures and subjected to western blot. Astrocytes produced EVs that were CD9 and CD63 positive (Fig. 3D). Astrocytes were next cultured from neonatal mice and media collected, pre-cleared, and subjected to nanoparticle tracking analysis. Media contained a singular prominent unimodal EV peak at ~136 nM (Fig. 3E). These results demonstrate that astrocytes produce CD9 positive EVs.

**Figure 4:**
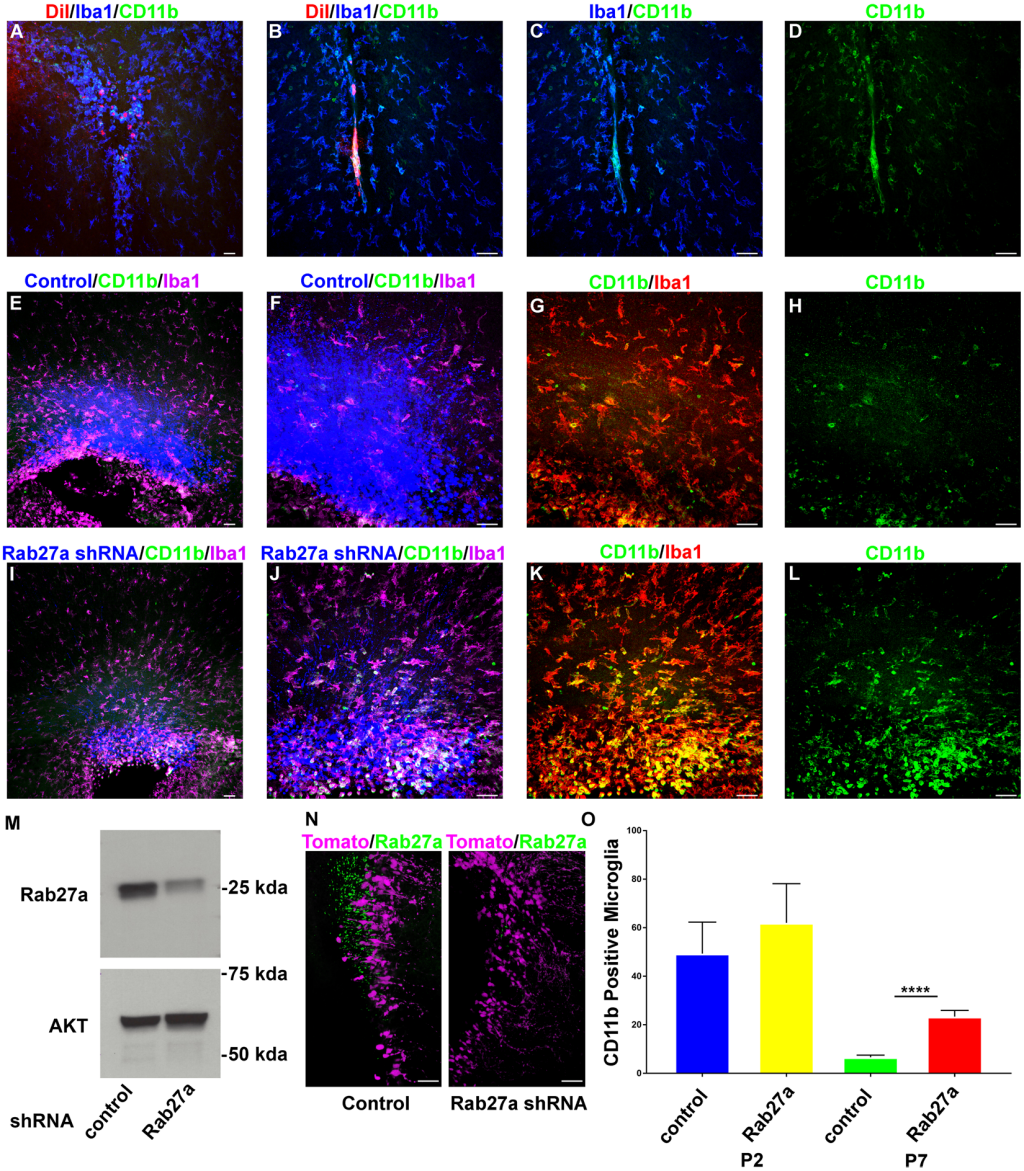
(**A**–**D**) Images of coronal brain sections showing DiI (red) labeled EVs taken up by IBA1 (blue) and CD11b (green) positive microglia. (**E,F**) Tomato and control plasmid (blue) dorsal SVZ electroporations stained for CD11b (green) and IBA1 (magenta) at P7, 10x (**E**), 20x (**F**). (**G**,**H**) 20x magnification of E showing CD11b (green) and IBA1 (red) (**G**) or CD11b (**H**). (**I**,**J**) Tomato and Rab27a shRNA plasmid (blue) dorsal SVZ electroporations stained for CD11b (green) and IBA1 (magenta) at P7, 10x (**I**), 20x (**J**). (**K**,**L**) 20x magnification of I showing CD11b (green) and IBA1 (red) (**K**) or CD11b (**L**). (**M**) Western blot of Rab27a following shRNA mediated knockdown. (**N**) Images of coronal section stained for Rab27a (green) following control or Rab27a shRNA and tomato (magenta) co-electroporation at P0 and sacrificed 48 hrs later. (**O**) Quantification of CD11b positive microglia at P2 and P7 following control or Rab27a shRNA electroporation. Data are represented as mean ± SEM. ****p < 0.0001 A, E, I scale bar, 100 μm. B–D, F–H, J–L, N scale bar, 50 μm.

Previous work has demonstrated that intraventricular injected NSC EVs are taken up by microglia. Therefore, intraventricular injection of DiI-labeled astrocyte EVs was performed. Similar to NSC EVs, astrocyte EVs were also taken up by CD11b/IBA1 positive immune cells having an elliptical morphology (Fig. 4A–D). Quantification revealed that 57.7% ± 7.30 of DiI labeled EVs colocalized with IBA1 positive microglia. Based on these results we hypothesized that the uptake of EVs by CD11b/IBA1 positive cells regulates the distribution of CD11b microglia within the SVZ. A loss of function approach was developed using shRNA to Rab27a which is required for exosome biogenesis. Four Rab27a shRNAs were examined, one of which was confirmed to knockdown Rab27a in mouse Neuro-2a neuroblastoma cells *in vitro* (Fig. 4M). tdTomato control or Rab27a shRNA encoding plasmids were co-electroporated into P0 SVZ NSCs. *In vivo* Rab27a knockdown was assessed 48 hrs after electroporation by immunohistochemistry. Rab27a shRNA resulted in a 93% reduction in Rab27a expression that was statistically significant (Rab27a shRNA = 7.64% +/− 1.45 vs. Control = 100% + /−29.34, P < 0.05, N = 4) (4N). Seven days post electroporation robust tdTomato expression was detected within dorsal SVZ NSCs having a basal radial projecting fiber that were nestin positive (Fig. 4E–H, data not shown). CD11b/IBA1 positive microglia were present within the SVZ and Rab27a knockdown resulted in similar numbers of CD11b/IBA1 positive microglia at P2. Electroporated dorsal NSCs give rise to astrocytes^12^. Between P2 and P7, the number of CD11b positive IBA1 positive microglia began to decrease (Fig. 4O). However, Rab27a increased the number of CD11b/IBA1 positive microglia in the SVZ (Fig. 4I–L,O). These results support a role for neonatal astrocyte EVs in regulating the number of CD11b positive microglia in the SVZ.

**Figure 5:**
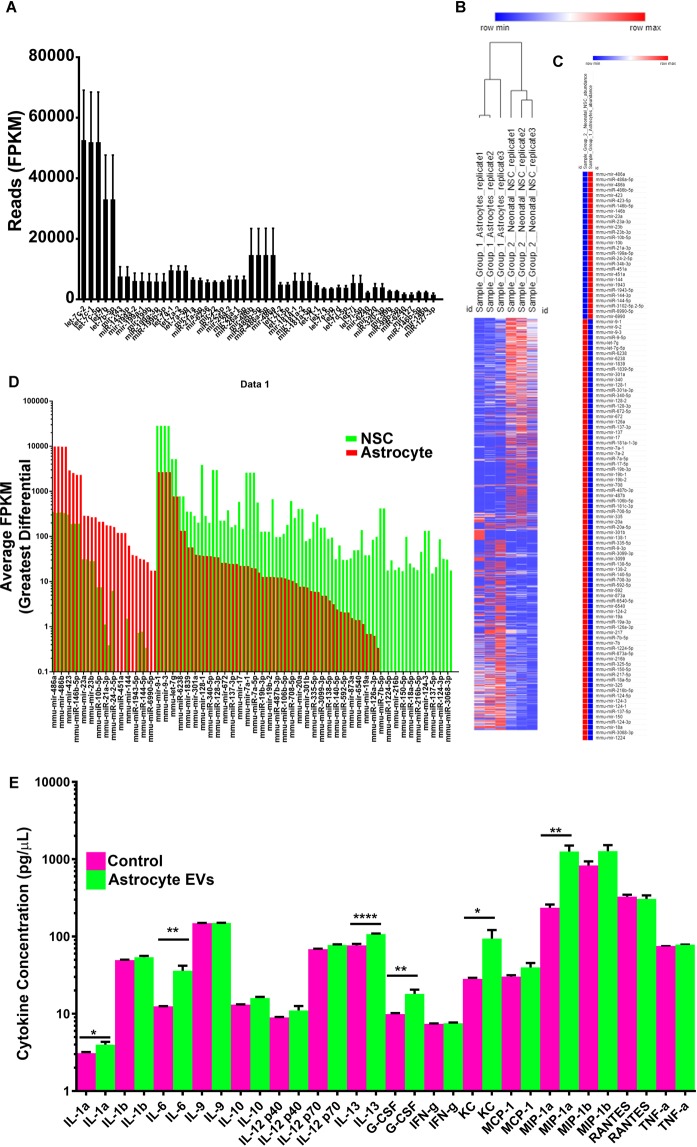
(**A**) Top most abundant miRNA reads from astrocyte EVs. (**B**) Hierarchical clustering analysis of small RNAs from neonatal NSC and astrocyte EVs. (**C**) Statistically significant up and down-regulated miRNAs. (**D**) Differential expression profile of individual miRNAs. (**E**) Cytokine profiles from microglia condition media. Data are represented as mean ± SEM. *p < 0.05; **p < 0.01; ****p < 0.0001.

Previous work demonstrated that NSC EVs contain immunomodulatory small RNAs and regulate a transcriptional network that induces microglia to increase the synthesis and release of cytokines. As an unbiased approach to begin to examine astrocyte EV functions, small RNA sequencing of astrocyte EVs was also performed. It was not surprising that neonatal astrocyte EVs contained many of the same miRNAs identified in neonatal NSC EVs including Let-7 family members (Fig. 5A). To determine the similarity of astrocyte EVs to NSC EVs, small RNA signatures determined by small-RNA sequencing were subjected to hierarchical clustering. Astrocyte EVs clustered with astrocyte EVs whereas NSC EVs clustered together (Fig. 5B). A small group of miRNAs were significantly differentially represented in astrocyte EVs and NSC EVs, many of which were several-fold differentially expressed (Fig. 5C,D). It was tempting to speculate that based on the conservation of immunomodulatory miRNAs in astrocyte EVs and the temporal induction of CD9, that astrocyte EVs may have immunomodulatory and morphogenic properties similar to neonatal SVZ NSCs. To test this hypothesis, EVs from neonatal astrocytes were used to treat cultured microglia. Astrocyte EVs stimulated a unique microglia cytokine profile (Fig. 5E). Taken together, these results demonstrate that neonatal astrocytes generate CD9 positive EVs that are targeted to and modify microglia.

## Discussion

Here we report the generation of an inducible CD9-GFP mouse. Tetraspanin proteins are useful for the isolation, characterization, and functional evaluation of EVs. A notable triad of exosome tetraspanin proteins includes CD9, CD63, and CD81^22–25^. Overlap between these markers exists, although each can also demarcate unique vesicle populations^25^. CD9 and CD63 are found throughout the secretory pathway including in lysosomes and the cell membrane but are enriched within MVBs^26^. CD63 fluorescent fusion proteins have been used for over a decade to study EVs. Notable examples are the use of CD63-GFP to demonstrate that classical morphogens such as Hedgehog and Wingless are transferred with EVs^27,28^. CD63-GFP has been used to study exosomes in mammalian EVs, including from mouse, rat, and human cells. For example, CD63-GFP was used to demonstrate transfer of EVs from T cells through an immunological synapse to antigen presenting cells^29^. More recently tag-GFP from copepods was fused to CD63 and used to create transgenic rats^30^. The expression of CD63-GFP is sustained from a ubiquitously activated CAG promoter (CAG-CD63-GFP)^30^. CAG-CD63-GFP was found in many tissues including within the heart, lung, and cortex, hippocampus and cerebellum of the brain. Moreover, CD63-GFP containing EVs could be isolated from biological fluids or from media conditioned from CD63-GFP expressing primary cell cultures^30^. A similar SOX2-CD63-GFP rat was generated although this model had a much more restricted developmental CD63-GFP distribution and was enriched in NSCs^31^. The TIGER mouse offers the primary advantage of combining ubiquitous expression with a CRE-inducible system and allows for cellular and temporal control of EV labeling. Expression therefore could be titrated to more cell types as demonstrated with CAG-CRE-ER^T2^ or fewer as occurred with Nestin-CRE-ER^T2^. Using CLARITY, robust *in vivo* labeling was demonstrated within a wide range of tissue types including spleen, heart, kidney, and brain. To our surprise, the subcellular distribution of CD9-GFP was frequently cell-type dependent. These results suggest that CD9 may label distinct EV subtypes, but this depends on the cell of origin.

A convergent cell-type labeled by CAG-CRE-ER^T2^ and *nestin*-CRE-ER^T2^ were astrocytes. Labeling within the brains of CAG-CRE-ER^T2^ mice was robust by P10 as evidenced by CLARITY whole brain imaging. Cells had a bushy appearance similar to astrocytes and when subjected to immunohistochemistry, labeled cells within the cortex were frequently positive for astrocyte markers GFAP or GS. These results led to the hypothesis that astrocytes from early cortical development release EVs. Astrocytes from the adult brain release EVs, however whether astrocytes release EVs during development was unknown. Here, cultured cortical astrocytes released EVs that were 136 nm in size and contained CD9 and CD63. CD9 expression was up-regulated within the cortex of the juvenile brain and into adulthood as quantified by western blot. Bioinformatic analysis confirmed astrocyte CD9 and CD63 expression which was confirmed by western blotting neonatal cortical astrocytes and cortical astrocyte EVs.

Microglia infiltrate the cortex after the first postnatal week. Neonatal NSC EVs can regulate microglia^4^. Since dorsal SVZ NSCs give rise to astrocytes, it was hypothesized that astrocyte EVs might also regulate microglia. The ability of astrocyte EVs to mobilize peripheral immune cells also supports this hypothesis^32^. In further support of this hypothesis, the majority of injected DiI labeled astrocyte EVs were taken up by CD11b positive IBA1 positive microglia. The possibility that other cell types may take up astrocyte EVs cannot be ruled out. In addition, by seven days post-electroporation, Rab27a knockdown increased the number of CD11b/IBA1 microglia. These results are surprising given the activated phenotype of microglia cultures exposed to NSC EVs^4^. As opposed to inducing an activated CD11b state, these results suggest those microglia having an activated state selectively take up EVs and that this uptake is associated with conversion to a non-CD11b state or dispersion from the SVZ.

To the surprise of the investigators, EVs from NSCs and astrocytes had considerable overlap, including members of the Let-7 family, yet clearly had a distinct signature. In relation to this signature, the effect of astrocyte EVs induced microglia cytokine profiles that differed from NSC EV induced responses. It is unclear at this time what accounts for the differential immune responses to EVs but could be related to the RNA signature present within the cell types. It would appear that molecules that function similar to PD-L1 or noncoding RNAs such as hY4 may be responsible for differences in immune responses^33,34^. Identifying differences in EV content and immune responses will prove critical in developing novel immunomodulatory strategies for regulating the neuro-immune axis.

## Methods

### Animals

The experimental protocols were approved by the Clemson University Institutional Animal Care and Use Committee. Experiments were performed according to guidelines set forth by the Clemson University Institutional Animal Care and Use Committee and NIH Guide for the Care and Use of Laboratory Animals. For primary cell culture experiments and for neonatal electroporations, pregnant CD-1 mice were obtained from Charles River Laboratories. The following strains were acquired from Jackson Laboratories and crossed to CD9 mice: Tomato, B6.Cg-Gt(ROSA)26Sortm9(CAG-tdTomato), Hze/J Stock No: 007909 Ai9; Nestin-CRE-ER^T2^, C57BL/6-Tg(Nes-cre/ERT2)KEisc/J, Stock No: 016261; CAG-CRE-ER^T2^, B6.129-Gt(ROSA)26Sortm1(cre/ERT2)Tyj/J, Stock No: 008463. Mice were housed under pathogen-free conditions with a 12-hr light/dark cycle. Pooled samples of both genders were used.

### Astrocyte culture

24 hrs prior to culture initiation, wells of a sterile 6 well plate were coated with 2 µg/mL of Poly-L-Lysine stock solution (Sciencell: Catalog #0413) in dPBS according to the manufacture’s instructions. Wells were then washed twice in nuclease free, sterile water prior to plating cells. P4 CD1 mouse pups were euthanized and dorsal cortices were removed. Using a scalpel, cortices were chopped into pieces, and placed into a 15 ml conical tube containing 2 mL of cold DMEM on ice. 400 μL of Trypsin/EDTA was added to the 2 mL of DMEM and placed in the incubator for 10 mins. Cells were triturated with three Pasteur pipettes fire polished to have decreasing bore sizes. 400 μL of trypsin inhibitor was added. Samples were then centrifuged at 200 × g for five mins. The supernatant was disposed of and the pellet was resuspended in 2 ml of warm DMEM for an additional centrifugation step of 200 × g. After decanting the warm DMEM, 10 mL of astrocyte media was added, and the pellet was resuspended. 10 mL of astrocytes in solution was added to a T75 flask containing fresh 15 mL of astrocyte media and pretreated with Poly-L-Lysine. After 4 days, the media was replaced. 7 days post-seeding, the flasks were rotated at 180 RPM for 30 min to remove microglia. The media was exchanged for fresh astrocyte media and rotated for an additional six hrs at 280 RPM to remove oligodendrocytes. The flask was shaken vigorously by hand for 1 min and media decanted. The remaining cells (astrocytes) were next trypsinized and split into 2 new Poly-L-Lysine treated T75 flasks using exosome-free astrocyte media. Four days later the media was changed. 7 days following the split, the enriched astrocyte cultures in the T75 flasks were trypsinized, counted, and seeded into wells. Cortical astrocytes were plated at 1.66 × 10^5^ cells per well in complete astrocyte culture media (2% Fetal Bovine Serum, 100 units/mL Pen-strep, Astrocyte Culture Media [Sciencell; Catalog #1801]). Cultures were place in a 37 °C incubator with 5% CO_2_. After 24 hrs, half of the culture media was replaced with fresh astrocyte culture media. 48 hrs after culture initiation, culture media was collected and used for luminex assays and extracellular vesicle isolation.

### Small RNA sequencing

RNA was isolated by Trizol extraction and SeraMir Exosome RNA column purification (Systems Biosciences). Samples were quantified using the Agilent Bioanalyzer small RNA assay. Libraries were prepared according to manufacturer’s protocol (Illumina small RNA preparation kit). Samples were subjected to 1 × 75 bp single-end reads at an approximate depth of 10–15 million reads per sample on an Illumina Hi-Seq. Aligned reads are analyzed with Banana Slug Analytics Platform (University of California at Santa Cruz).

### CLARITY

Tissues were fixed in 4% paraformaldehyde overnight at 4 °C, and subsequently washed for 15 mins in PBS three times at room temperature. Samples were incubated overnight in a hydrogel monomer solution made up with the X-Clarity Hydrogel Solution kit (Logosbio: Catalog #C1310X). The following day, tissue embedded in the hydrogel monomer was placed within the X-Clarity Logos Polymerization System (Logosbio: Catalog #C20001) for three hrs. Tissues were then briefly washed in PBS at room temperature and placed into the X-Clarity Tissue Clearing System (Logosbio: Catalog #) filled with Electrophoretic Tissue Clearing Solution (Logosbio: Catalog #C13001). Tissue cleared within 8 hrs. The tissue was then washed in room temperature PBS overnight, incubated in X-Clarity Mounting solution (Logosbio: Catalog #C13101) at 37 °C for 30 mins to an hr, and imaged using a Leica SP8 Multiphoton Confocal.

### EV Isolation and labeling

Primary astrocyte cell culture media was subjected to differential centrifugation. Samples were pre-cleared to remove dead cells and debris by performing a 300 × g centrifugation for 10 mins and the supernatant was transferred to a new tube and centrifuged at 2,000 × g for 10 mins. Finally, the media was subjected to a 100,000 × g spin at 4 °C in a Beckman Coulter Optima MAX-XP with a TLA 100.3 rotor for 90 mins (P100 fraction). Centrifuge tubes were weighed and balanced with dPBS prior to centrifugation. Pellets were re-suspended in 25 μL 2% SDS in RIPA buffer containing protease and phosphatase inhibitor cocktail for western blot analysis or 50 μL dPBS for nanoparticle tracking analysis or microglia treatment. Astrocyte EVs were incubated with 0.2 μm prefiltered DiI (ThermoFisher Scientific, V22889) at a 1:1000 dilution (1 μM) for 10 min at RT. Samples are placed into 2.0 mL of dPBS and recentrifuged at 100,000 × g at 4 °C for 30 min. Supernatant was removed and fresh sterile dPBS was added to EVs and vortexed. A Hamilton Syringe with Neuros adapter was loaded with 1–2 μL of DiI-labeled EVs (5.52–11.4 × 10^7^ EVs). P0 CD1 mouse pups are anesthetized on ice for 5 min until pup is not ambulatory. Injections were performed by locating the sagittal suture of the developing skull along the midline of the brain. Hamilton NeurosSyringe needle was placed near the rostral portion of the brain at the midline. The needle was inserted approximately 1 mm laterally and 0.5 mm caudally into the lateral ventricle approximately 2 mm deep. 1–2 μL of DiI-labeled EVs (5.52–11.4 × 10^7^ EVs) were injected into the lateral ventricles. Pups were immediately placed on heating pads for 5 min. Once ambulatory, pups were placed with mothers and sacrificed 1–7 days later.

### Immunohistochemistry

Tissue was harvested and fixed in 4% PFA overnight at 4 °C. Tissue was washed for 1 hr at room temperature in PBS, mounted in 3% agarose, and sectioned into 200 μm thick sections with a Leica VTS 1000 vibratome. Sections were subsequently blocked for an hr in blocking solution (PBS, 0.1% Tween-20, 0.1% Triton-X 100, and 2% BSA). Sections were washed with 0.1% Tween-20 in PBS three times for a total of 15 mins. Sections were incubated in antibody solution (0.1% Tween-20 and 2% BSA in PBS) along with primary antibodies overnight at 4 °C. The following day, slices were washed for 30 min with three exchanges of wash buffer. Sections were incubated in antibody solution with secondary antibodies for 1 hr at room temperature or overnight at 4 °C. Following secondary antibody incubation, sections were washed for 10 min in wash buffer for a total of five times and once in PBS for 10 min prior to being mounted. Antibodies used were GFAP (Cell Signaling: Catalog #12389), IBA1 (Novus Bio: Catalog #NB100-2833), CD11b (Bio-Rad: Catalog #MCA711G), and Glutamine Synthetase (Sigma Aldrich: Catalog #G2781), Rab27a (Cell Signaling: Catalog# D7Z9Q).

### Quantification of microglial cells

The number of CD11b + microglia were quantified in FIJI (Image J) using the cell counter plugin. Confocal Z sections were loaded in FIJI, individual colored channels split, and converted into RGB images. CD11b + microglia were quantified by hand and tracked by the cell counter plugin.

### Quantification of DiI uptake by microglia cells

Images were loaded onto FIJI (Image J). Images were converted to a composite image. Z sections, selected based on DiI and IBA1 staining, were max Z projected. Confocal color channels were split. The DiI and IBA1 channels were thresholded, 0–255, to account for background. The colocalization threshold feature was used to produce a colored image showing colocalization. The image was then converted to an 8-bit image, inverted, and thresholded to visualize only co-localized particles. Particle analysis was then used to quantify the number of DiI and IBA1 colocalized particles. Particle analysis was also performed on the thresholded DiI image to quantify the total number of DiI particles. The percentage of co-localized DiI particles was in relation to the total number of DiI particles^4^. Three Z sections per image were used to calculate the average percentage of colocalized particles. These values were then inserted into GraphPad Prism 7.04 to generate a graph.

### Quantification of Rab27a Knockdown

20x confocal images were loaded into FIJI software and max Z Projected. Individual channels were split and subjected to automated particle analysis using the particle analysis plugin. Particle size was set from 1 to infinity and the mean number of particle sizes and standard error mean were documented.

### Electroporations

Electroporations were performed as described previously^16^. Briefly, P0 mice were injected with CAG-CRE, CAG-tdTomato, and/or Rab27a shRNA plasmid into the lateral ventricle of the subventricular zone. Pups were then electroporated with a BTX ECM 830 Square Wave Pulse generator and tweezertrodes (Harvard Apparatus). Electroporations were targeted to dorsal SVZ neural stem cells by placing the positive electrode on the lambda suture of the skull.

### Nanoparticle tracking analysis

Samples were shipped to the Nanomedicine Characterization Core facility in the Center for Nanotechnology in Drug Delivery at the University of North Carolina (UNC) Chapel Hill. Samples were prepared in a laminar flow hood and thawed at RT. Sample dilutions were based on an initial run with PBS, 10 mM salt. Samples were loaded onto a pre-cleaned and pre-warmed Nanosight NS 500 nanoparticle characterization system (NanoSight, UK) equipped with a 532-nm laser and a 565-nm long pass filter. The Nanosight NS 500 was calibrated with 100 nm polystyrene latex microsphere standards (Nanosight, UK), and readings were acquired at 23.3 °C. Values were entered and graphed using GraphPad 7 Prism software.

### Long range PCR

DNA was isolated using the Qiagen DNeasy Blood and Tissue Kit (250) (Catalog #69506). Briefly, samples were placed into a microcentrifuge tube with buffer ATL and Protein Kinase A. Samples were incubated at 56 °C and vortexed every hr until tissue became transparent. Once the tissue is transparent, buffer AL and ethanol were added to the samples. The mixture was then pipetted into a DNeasy mini spin column and centrifuged at 6,000 × g for one min. The column is then placed into a new collection tube with AW1 and centrifuged at 6,000 × g for one min. The collection tube is replaced again, buffer AW2 is added to the sample, and centrifuged at 20,000 × g for three mins. To ensure that the sample is dry, the flow through is removed and the sample is centrifuged again for three mins at 2,000 × g. The DNeasy mini spin column is then placed into a 1.5 mL microcentrifuge tube and 50 μL of buffer AE is pipetted directly onto the membrane. The samples are then incubated at room temperature for 5 mins and sequentially centrifuged at 6,000 × g for one min. This step is repeated using the flow-through. The samples were then quantified with a NanoDrop spectrophotometer to ensure that the DNA concentration was at least 10. Samples are stored at −20 °C. Using the Qiagen Long Range PCR Kit a master mix consisting of 5 μL Long Range PCR Buffer, 2.5 μL dNTP mix, 10 μL 5X Q Solution, 0.2 μL of each primer, 0.4 μL LongRange PCR Enzyme Mix, and enough RNase Free Water to make the mix 50 uL total per reaction. 49 μL of master mix was added to a PCR tube along with 1 μL of DNA. The samples were then placed into a thermocycler. Following amplification of the DNA, samples were loaded into a gel with 1X Blue Juice and run at 100 V for 20–30 mins. The primers for 5′ targeting are 5′-CGCCTAAAGAAGAGGCTGTG-3′ and 5′-TCATCAAGGAAACCCTGGAC-3′ and for 3′ targeting are 5′-AACAAGCACTGTCCTGTCCTCA-3′ and 5′-TAGTTGCCAGCCATCTGTTGTT-3′.

### Western blot

Samples were lysed in RIPA buffer containing 2% SDS, and protease and phosphatase inhibitor cocktail. Samples were placed on ice, sonicated, centrifuged and supernatants placed in a fresh tube. Protein samples diluted in Laemmli were run on 10% polyacrylamide gels and transferred to PVDF membranes using a Bio-Rad PROTEAN gel system. Membranes were blocked in TBS, 0.1% Tween-20, and 5% milk and incubated with the following antibodies: CD9 (Systems Biosciences, 1:1,000), CD63 (Systems Biosciences, 1:1,000), Rab27a (Cell Signaling, 1:1,000), pan-AKT (1:1,000, Cell Signaling). Horseradish peroxidase conjugated secondary antibodies were used at a concentration of 1:3,300. Membranes were incubated with Enhanced Chemiluminescent Substrate (Pierce) and signal detected with Amersham Hyperfilm (GE Healthcare).

### N2a Cell culture and transfections

Neuro-2a cells (CCL-131, ATCC) were plated into 6-well plates at a density of 100,000 cells/well and allowed to attach overnight. Media was refreshed 30 mins prior to transfection. For transfection, 1 μg plasmid (CAG-GFP control or one of four distinct shRab27a plasmids) was incubated at room temperature in serum-free DMEM with 3 μL PolyJet (Signagen) for 15 mins. After 24 hrs, the cells were lysed, concentration determined by BCA protein assay (Sigma Aldrich) and prepared for Western Blot.

### Fibroblast culture

Mouse tail snips were used for isolation of primary fibroblasts. Tails were cut into small pieces using scissors and placed in a conical tube with a solution of complete DMEM and collagenase. Pieces were placed in a shaking incubator at 37 °C for 90 mins, followed by manual grinding of tissue pieces. After tissue digestion, the solution was put through a cell strainer, centrifuged at 1,000 × g for 5 mins and cells were plated on standard tissue culture plates in pre-warmed complete DMEM.

### Genotyping PCR

DNA samples were incubated in 75 μL 50 mM NaOH buffer at 50 °C until tissue is transparent. After incubation, 75 μL of 100 mM Tris-HCl was added to samples. Samples were stored at −20 °C. The following primers were used: Tomato (AG GGA GCT GCA GTG GAG TA, CG AAA ATC TGT GGG AAG TC and GGC ATT AAA GCA GCG TAT CC, CTG TTC CTG TAC GGC ATG G), CRE (GCG GTC TGG CAG TAA AAA CTA TC, GTG AAA CAG CAT TGC TGT CAC TT and CTA GGC CAC AGA ATT GAA AGA TCT, GTA GGT GGA AAT TCT AGC ATC ATC C).

### Microglia culture and EV treatments

24 hr prior to plating microglia, 6 well plates were coated with 10 μg/mL poly-L-lysine. Microglia isolated from CD1 mice were were plated in complete media (2 ng/mL TGF-b2, 100 ng/mL IL-34, 1.5 μg/mL cholesterol, 100 units/mL penicillin-streptomycin, Glutamax, and microglia media [Sciencell, 1901]) and allowed to adhere for 10 min at RT. For cytokine assays, 1.66 × 10^5^ cells per well on 6 well plates and treated 24 hrs later. EVs were isolated from astrocytes plated at a density of 1.66 × 10^5^ cells per well of a 6 well plate in 3 mLs per well. 3 mLs of astrocyte conditioned media was subjected to EV isolation, resuspended in 50 μL media (27.6 × 10^8^ EVs in 50 μL), and added to microglia cultured in 3 mLs of media per well in 6 well plates. 24 hrs later, media was collected and depleted of all EVs and cytokine assays performed.

### Cytokine assay

Cytokine concentrations were quantified with the cytokine multiplex assay from Bio-Rad as recently described^4^. Wells of a 96 well filter plate were loaded with 50 mL of prepared standard solution or 50 mL of cell-free supernatant and incubated with the Bio-Plex Pro mouse 23-plex assay from Bio-Rad at ±800 rpm for 30 min in the dark at RT. Wells were vacuum-washed three times with 100 mL wash buffer. Samples were then incubated with 25 mL of biotinylated detection antibody at ±800 rpm for 30 min at RT in the dark. After three washes, 50 mL of streptavidin-phycoerythrin was added to each well and incubated for 10 min at ±800 rpm at RT in the dark. After a final wash, the beads were resuspended in 125 mL of sheath buffer for measurement with the Luminex 200 (Luminex, Austin, TX).

### Statistics

Statistics were performed with Prism software (version 7; GraphPad). Significance was calculated using unpaired t tests or one-way ANOVA with Tukey’s multiple comparisons test. All data are presented as mean ± SEM. Heat maps and hierarchical clustering analysis was performed using the University of California at Santa Cruz Banana Slug Analytics Platform.

## Supplementary information

Supplementary Figures
