# Supplementary material for: A transgenic inducible GFP extracellular-vesicle reporter (TIGER) mouse illuminates neonatal cortical astrocytes as a source of immunomodulatory extracellular vesicles
Source: Sci Rep. 2019 Feb 28;9:3094. doi: 10.1038/s41598-019-39679-0 (PMC6395689; doi:10.1038/s41598-019-39679-0)

Supplementary Title Page

Title: A transgenic inducible GFP extracellular-vesicle reporter (TIGER) mouse illuminates neonatal cortical astrocytes as a source of immunomodulatory extracellular vesicles

Victoria N. Neckles[1], Mary C. Morton[1], Jennie C. Holmberg[1], Aidan M. Sokolov[1], Timothy Nottoli[2], Don Liu[3], David M. Feliciano[1]*

1. Department of Biological Sciences, Clemson University, Clemson, SC 29634-0314, USA.

2. Yale Genome Editing Center, Department of Comparative Medicine, Yale School of Medicine, PO Box 208016, New Haven, CT 06520-8016, USA.

3. Charles River Laboratories, 251 Ballardvale Street, Wilmington, MA 01887

*Lead Contact; To whom correspondence should be addressed: David M. Feliciano, Ph.D., Department of Biological Sciences, Clemson University, Clemson, SC 29634-0314, USA. Tel: 864.656.2328 FAX: 864.656.0435;

E-mail: dfelici@clemson.edu

Supplemental Figure Legend

Supplemental Figure 1. A. Schematic diagram TIGER mouse prior to and after CRE mediated recombination. **B.** Neuro-2a cell transfected with CAG-tomato (red), CAG-CRE, and CD9-GFP (green) targeting plasmid and counterstained with TOPRO (blue). **C.** 5' ROSA26 targeting long range PCR assay. Black arrow indicates clones positive for 5' long range amplicon. **D.** 3' ROSA26 targeting long range PCR assay. Black arrow indicates clones positive for 3' long range amplicon. **E.** Founder mice generated litters with proper mendelian ratios and without defects. **F.** DNA gel of PCR using Turbo-GFP primer. **G.** DNA gel of Tomato and CRE PCR amplicons following crosses to generate appropriate genotypes.

Supplemental Figure 2. A. Schematic diagram of electroporation at P0. **B.** Image of CD9-GFP electroporated cells surrounding the SVZ. **C.** Tracing of the cells from B. accounting for tomato expression to illuminate the soma. **D.** Schematic of plasmid used for electroporation along with the mouse genotypes for E. **E-G.** 5, 10, and 20x zoom-2 coronal sections at P2 following electroporation of CRE and counterstained with TOPRO (blue). Note recombination demonstrated by tomato (red) and the subcellular localization of CD9-GFP (green) surrounding tomato positive cells.

Supplemental Figure 3. A. Schematic diagram of genotypes of cross between TIGER mice, tomato mice, and nestin-CRE-ER^{T2} mice. **B, C.** CD9-GFP (green) and tomato (red) expression following tamoxifen injection and subsequent expression within the RMS-OB at 5x (B) and 20x (C). **D, E.** CD9-GFP (green) and tomato (red) expression following tamoxifen injection and subsequent expression within the cerebral cortex at 5x (D) and 20x (E). **F, G.** CD9-GFP (green) and tomato (red) expression following tamoxifen injection and subsequent expression within the Hippocampus at 5x (F) and 20x (G). **H, I.** CD9-GFP (green) and tomato (red) expression

following tamoxifen injection and subsequent expression within the cerebellum at 5x (H) and 10x (I).

Supplemental Western Blots. Uncropped western blots

Supplemental Figure 1

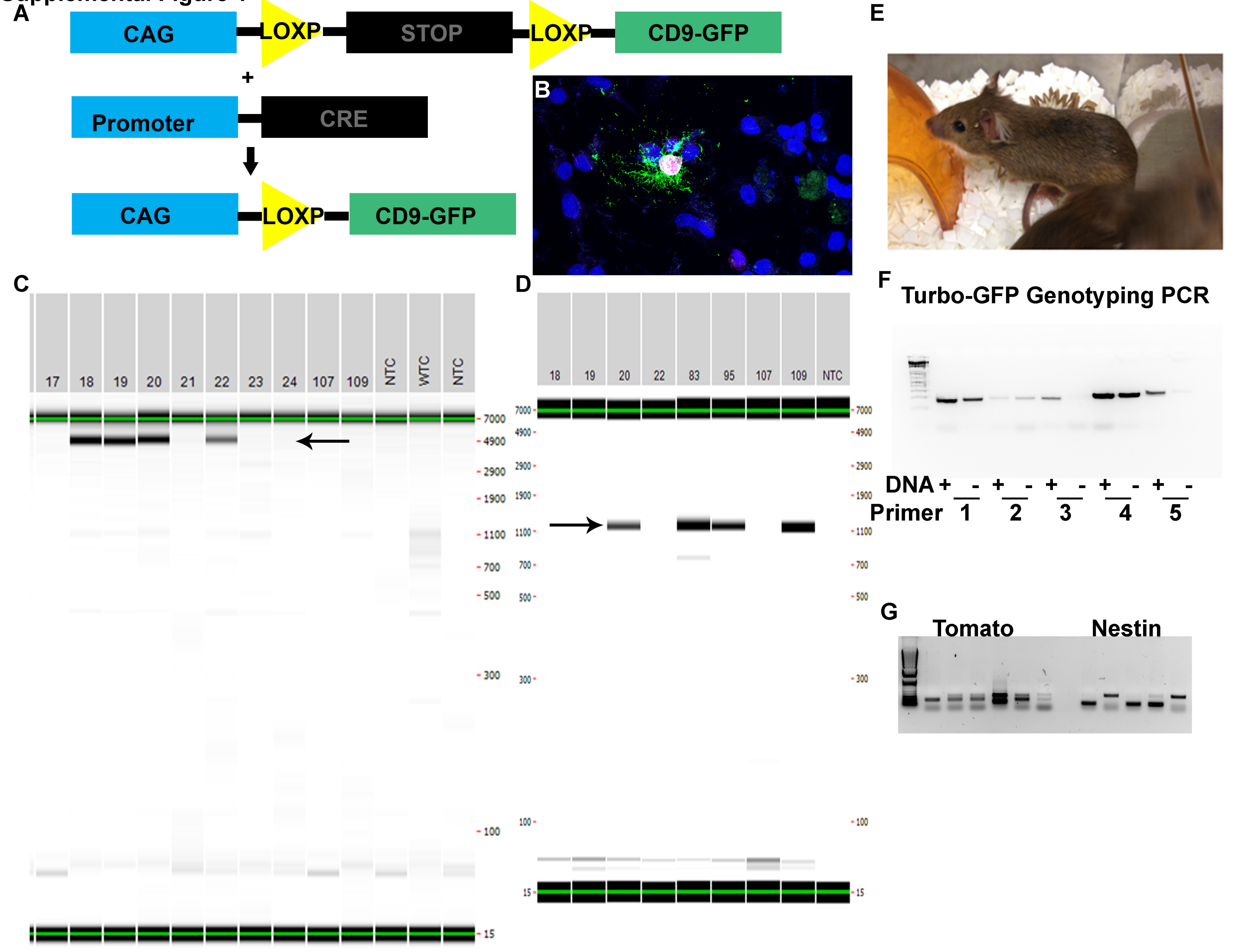

Supplemental Figure 2

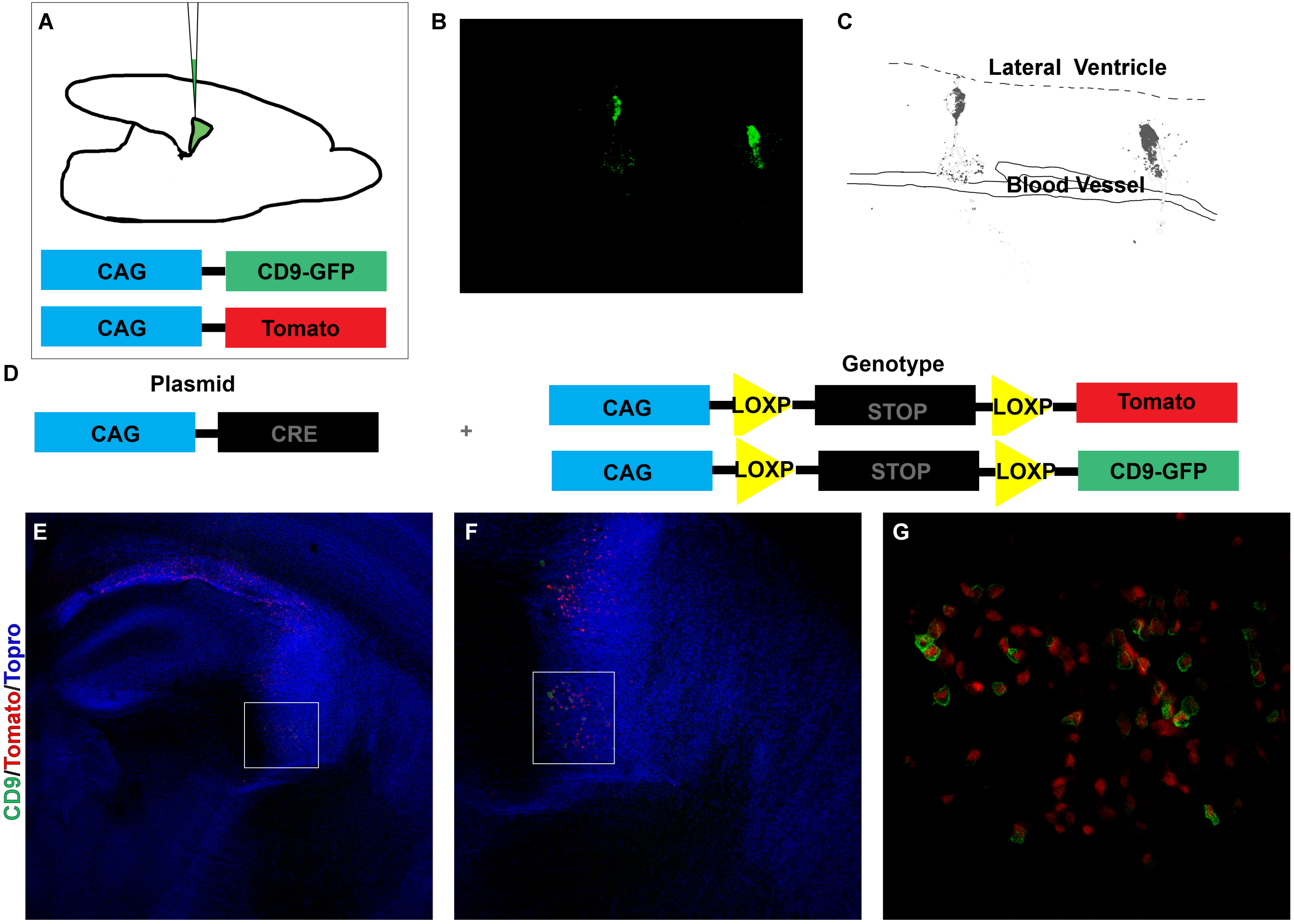

Supplemental Figure 3

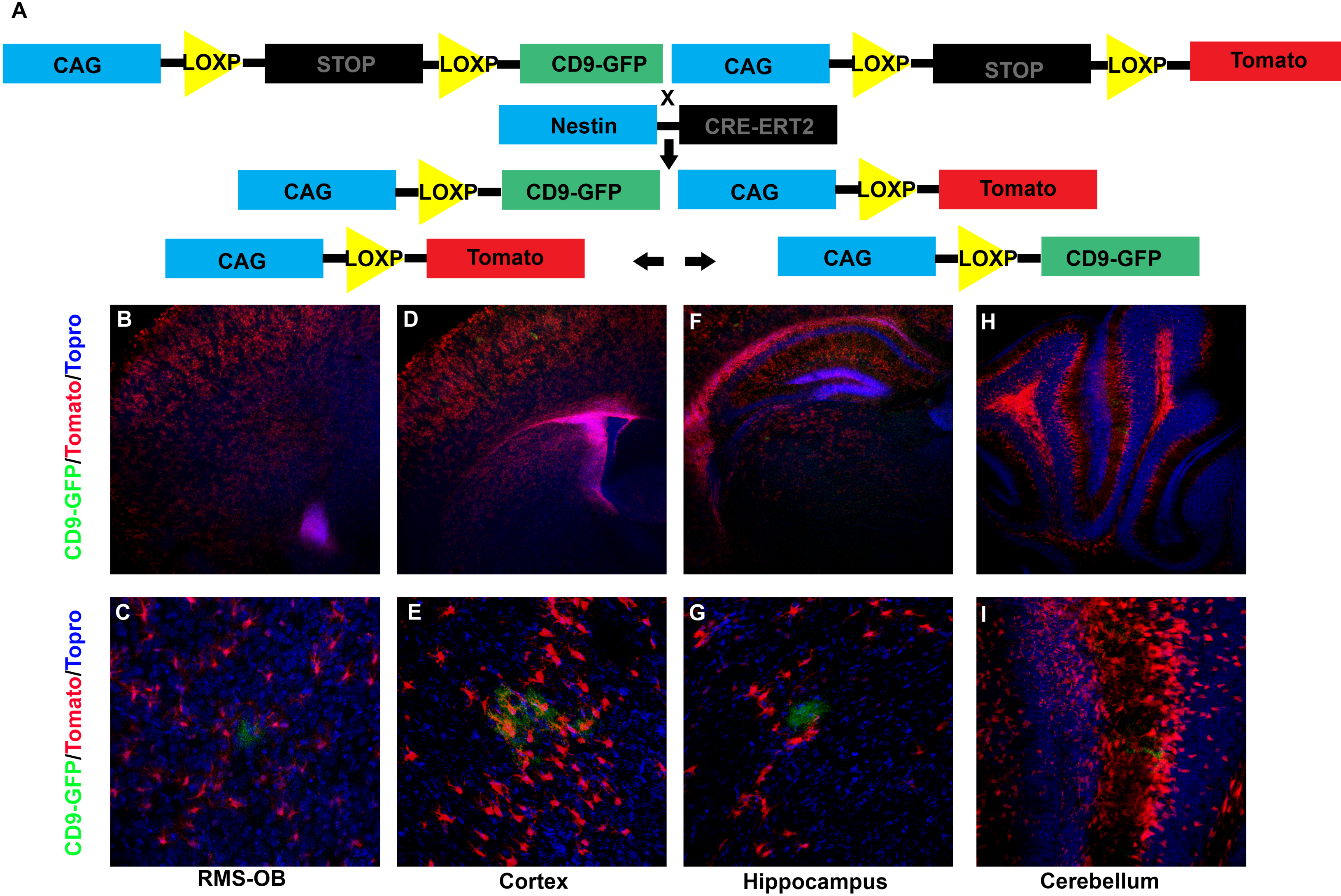

Supplemental Westerns

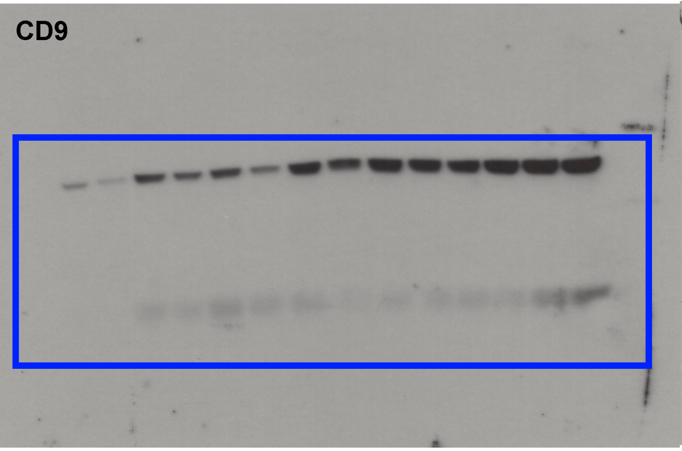

Long Exposure

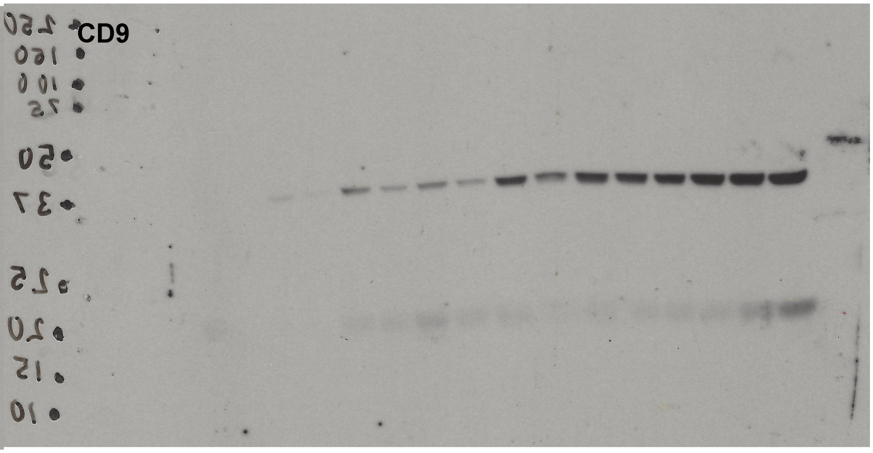

Short Exposure

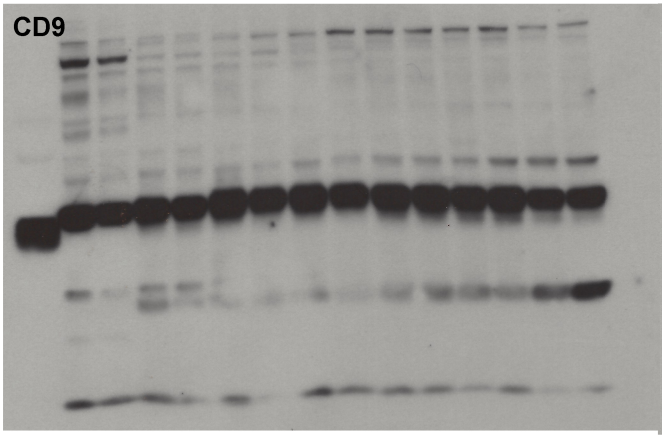

Long Exposure

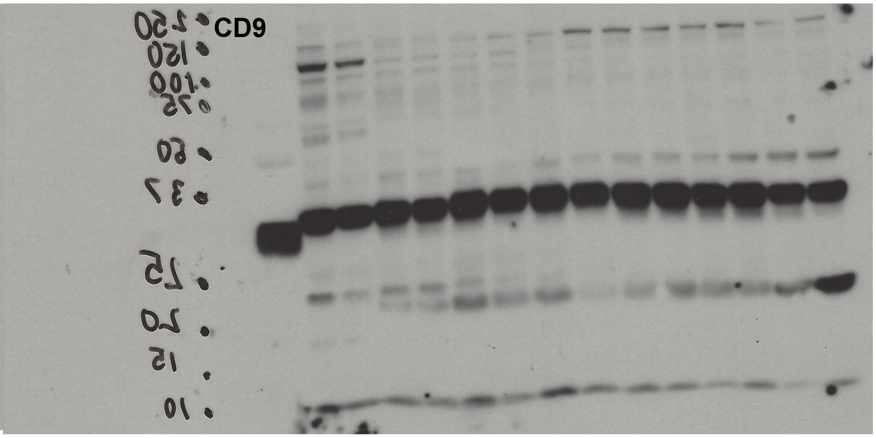

Short Exposure

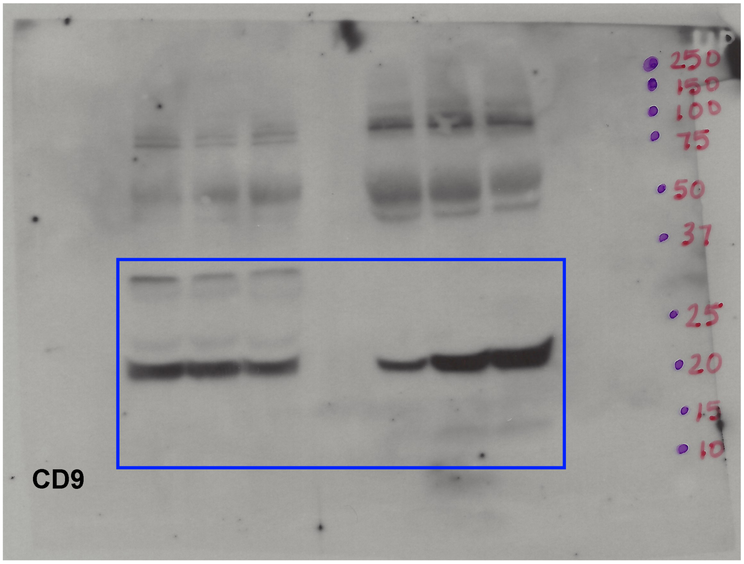

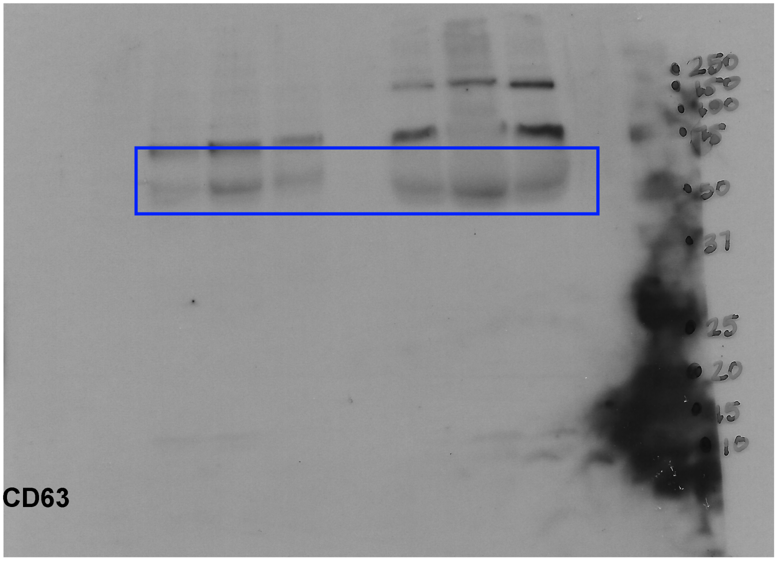

Supplement: Supplementary file 1 — Supplementary Figures [file 41598_2019_39679_MOESM1_ESM.pdf]
